# Bending Strength of Polyamide-Based Composites Obtained during the Fused Filament Fabrication (FFF) Process

**DOI:** 10.3390/ma15145079

**Published:** 2022-07-21

**Authors:** Michał Mazurkiewicz, Janusz Kluczyński, Katarzyna Jasik, Bartłomiej Sarzyński, Ireneusz Szachogłuchowicz, Jakub Łuszczek, Janusz Torzewski, Lucjan Śnieżek, Krzysztof Grzelak, Marcin Małek

**Affiliations:** 1 Institute of Robots & Machine Design, Faculty of Mechanical Engineering, Military University of Technology, 2 Gen. S. Kaliskiego St., 00-908 Warsaw, Poland; michal.mazurkiewicz@student.wat.edu.pl (M.M.); katarzyna.jasik@student.wat.edu.pl (K.J.); bartlomiej.sarzynski@student.wat.edu.pl (B.S.); ireneusz.szachogluchowicz@wat.edu.pl (I.S.); jakub.luszczek@wat.edu.pl (J.Ł.); janusz.torzewski@wat.edu.pl (J.T.); lucjan.sniezek@wat.edu.pl (L.Ś.); krzysztof.grzelak@wat.edu.pl (K.G.); 2 Institute of Civil Engineering, Faculty of Civil Engineering and Geodesy, Military University of Technology, 2 Gen. S. Kaliskiego St., 00-908 Warsaw, Poland; marcin.malek@wat.edu.pl

**Keywords:** additive manufacturing, three-point bending, polyamide-based composites, fused filament fabrication

## Abstract

The research shows the comparison between two types of polyamide-based (PA) composites and pure, base material. The conducted analysis describes how the additions of carbon fibers and glass microbeads affect the material’s properties and its behavior during the bending tests. All samples have been tested in the three main directions available during the FFF process. To extend the scope of the research, additional digital-image-correlation tests and fracture analyses were made. The obtained results indicated a positive influence of the addition of carbon fibers into the material’s volume (from 81.39 MPa in the case of pure PA to 243.62 MPa in the case of the PA reinforced by carbon fibers).

## 1. Introduction

Polyamide-based materials, due to their favorable mechanical properties, have the interest of various industrial sectors, including automotive, aviation, and biomedical ones. They are one of the most popular construction materials in many types of industries. PA materials are characterized by relatively high-performance properties such as strength, hardness, stiffness, fatigue, abrasion resistance, and good friction properties because of the low friction coefficient value. Due to their properties, PA materials are increasingly used in additive-manufacturing (AM) technology [1,2,3]. Parts obtained via AM, made of PA, are characterized by high durability and thermal and chemical resistance; additionally, they can be further processed by the means of subtractive technologies. These materials allow obtaining a good surface quality; hence, they are used not only for the production of prototypes but also as ready-to-use parts: gears, clamps, slide bearings, spacing spools, casings, or bushings [4].

In the case of AM technologies, PA-based materials are broadly used in the fused filament fabrication (FFF) and selective laser sintering (SLS) methods [5,6,7,8,9]. By means of those two methods, it is possible to obtain the geometrically complex parts and nonstandard components, which are characterized by relatively good performance properties and their production is relatively inexpensive in the case of low-volume production [10,11,12,13,14].

The most frequently used types of PA material in the FFF and SLS technologies are PA6, PA11, and PA12 [15,16,17]. In the case of the SLS technology, PA-made parts have a form of sintered particles, while in the FFF technology, the material is deposited as extruded lines. The connection between powder particles and extruded lines has a form of adhesion joints [18,19]; hence, the semicrystalline character of the PA polymers [20] is better exposed in parts obtained by means of the FFF technology.

An important feature of PA materials that can affect the AM parts’ properties is their hygroscopicity (water absorption from the air). Dry polyamide becomes brittle, and its impact strength is worse, while its tensile strength increases. In the opposite situation, the elasticity and impact strength increase, but at the same time, the tensile strength significantly reduces. In practice, when the filament absorbs water (which may constitute approx. 10% of its mass), it affects the adhesion of the part to the substrate plate as well as the adhesion between layers, to finally increase the surface roughness of the outline shells of AM parts. Additionally, such a phenomenon could foster nozzle clogging or a “bubbling effect” caused by overheating the moisture in the filament volume [21]. Another important issue related to PA-based materials is their proneness to warping, distortion, and worse possibility to obtain good-quality shapes in comparison to other available polymers dedicated for AM. Nowadays, with many available devices in the market, this drawback is not as significant as a few years ago, but still, it is a serious challenge, especially in the case of more geometrically complex parts. The basis of this phenomenon is related to the shrinkage stress generated during the crystallization process [22]. The characteristics of the AM technology based on layer-by-layer manufacturing cause an asynchronous volumetric shrinkage, with additional regularity in the arrangement of the molecular chains which, as a result, aggravates the shrinkage of the AM part [23]. The newest trends in PA-based material production for AM lead to weakening the crystallization abilities and hindering the regular arrangement of molecular shapes [24].

From the beginning of polymer manufacturing, researchers have tried to optimize the chemical composition of polymers to obtain the desired properties. An increase in an exact property (i.e., mechanical) worsens other types (electrical or thermal); hence, there are many different modifications of polymers that allow balancing between different material properties. Shirvanimoghaddam et al. [25] reviewed a state-of-the-art research work related to the optimization of polypropylene-based (PP) materials’ properties. On the one hand, the authors described the most popular, conventional methods of modification methods (elastomer additions, additional copolymerization, nucleating agents, fillers, etc.).

However, on the other hand, they highlighted some less conventional methods:

- Annealing of the composites as a post-treatment technique;
- The use of ultrasound energy during the processing;
- The potential of advanced fillers such as graphene, CNT, boron nitride, MXene, MoS2, and MOFs;
- Using pretreatment or post-treatment techniques;
- Enhancing the interfacial bonding.

Similarly to PP, the properties of PA-based materials can be selectively altered by using additives or fillers in order to obtain specific material characteristics. The resulting blends allow for improvements in the mechanical, thermal, and electrical properties over the typical properties of the base material. The most common additives are reinforcement fibers (carbon, aramid, and glass), plasticizers, and stabilizers. In many cases, the performance properties of the PA-based composites with fiber addition are greater than the pure, base material [26,27,28].

On the one hand, Rahim et al. [29] used the PA12 material combined with bioceramic fillers, which improved the tensile strength and stiffness of pure PA12, and on the other hand, Liao et al. [30] revealed the improvement in the mechanical and thermal properties PA 12 polymer reinforced with carbon fibers during the FFF process. Different research paths of the mentioned research [31,32] independently allowed for an improvement in the material properties. Additionally, the authors of [32] proved that the addition of 10% carbon fibers to the PA12 matrix may result in a noticeable increase in bending strength, as well as an increase in other performance parameters. A positive influence of using fibers as an addition to the PA-based materials was proven by Chacón et al. [31] who registered a very good influence of glass fibers in the PA composite samples during the three-point bending tests.

In the available literature, there are many results of some exact PA materials or PA-based composites [32,33,34,35,36]. A kind of approach based on a comparison of different PA materials and AM technologies is also available there. A good example is the work of Türk et al. [37], where the authors characterized the mechanical properties of the obtained samples (Young’s modulus, tensile yield point, and flexural-creep modulus). They used samples obtained by the SLS and compared the results with the samples made via the FFF process with the use of ABS. None of the reviewed papers include data on how each addition affects the material properties, which could be helpful to properly describe the influence of an exact addition in comparison to the pure, base material. Hence, this study investigates the influence of using additions (carbon fibers and glass microbeads) in the PA-based material on the strength properties during three-point bending tests. To extend the scope of the research, samples for each material were tested in three different printing directions. Additionally, the fracture mechanism during three-point bending testing was described by means of a macrostructural investigation. Such an analysis would be helpful to determine a proper application of AM parts made of each PA-based material, not only as temporary spare parts, but also as products for the final usage.

## 2. Materials and Methods

The materials used in this examination included pure polyamide PA6 (PA6 Neat Black) and two composites based on polyamide—PA6 GK10 and PA6 CF15. It has to be mentioned that the word “pure” in the case of PA6 NB denotes a lack of fibers, ceramics particles, etc. The base material is a blend with introduced additions to reduce warpage during the AM process. All materials were delivered by the Spectrum Filaments company (Spectrum Filaments, Pęcice, Poland). PA6 GK10 is a glass-microbead-doped (10% of the total volume of the material) composite. This type of reinforcement improves the temperature resistance and thermal insulation and, furthermore, lowers the influence of humidity on the mechanical properties [38]. PA6 CF15 is a polyamide-based composite which is carbon fiber (CF)-doped (15% of the total volume of the material). The reinforcement lowers hygroscope features and the material shrinkage during the process of extrusion [39] compared to pure PA6. The properties of all studied materials are presented in Table 1.

The FFF technology was used for AM of the samples dedicated for bending tests. The production process was performed by means of a 3DGence Double P255 device (3DGence, Katowice, Poland). All samples of the given material were produced in one process with the additional raft to improve adhesion features. The FDM process production was prepared with the use of 3DGence Slicer 4.0 (3DGence, Katowice, Poland). The FDM process parameters are shown in Table 2.

**Figure 1:**
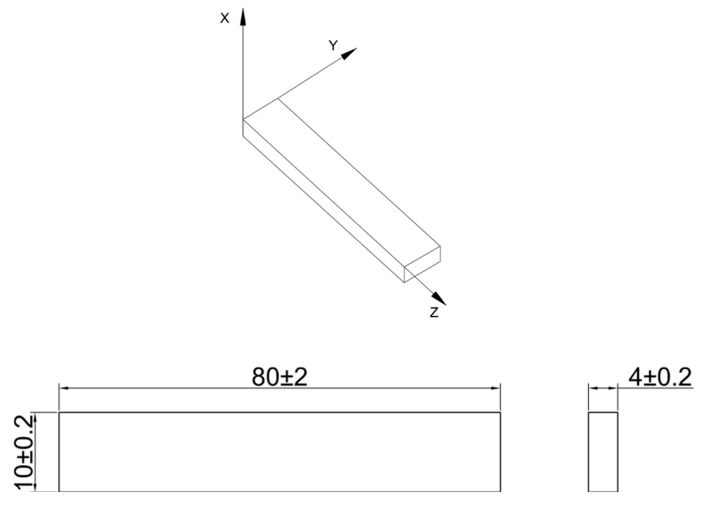
Axes of the test samples and geometric dimensions.

The 3D models of the samples were designed based on the PN-EN ISO 178, by means of CAD software Autodesk Inventor (Autodesk Inc., San Rafael, CA, USA). Apart from testing three different materials, the influence of the samples’ orientation during the AM process on the bending strength was also taken into account. Strength tests were performed for five samples for each of the directions: X, Y, and Z. The designation of the axes along which the printing directions were planned is presented in Figure 1. The specimens were investigated in the as-built state.

**Figure 2:**
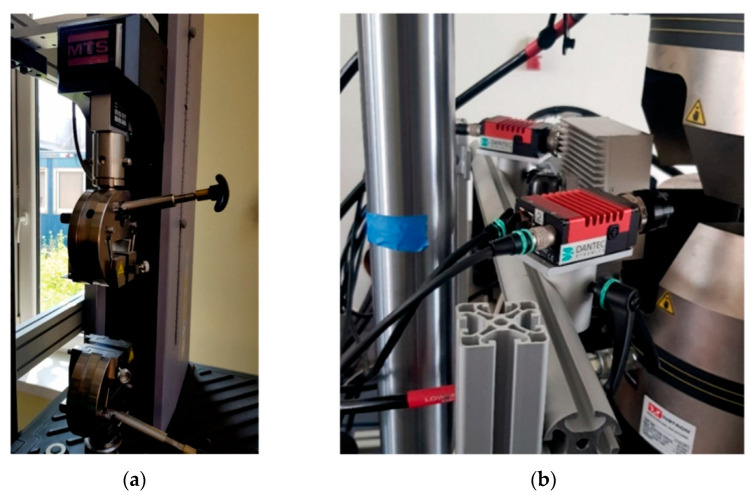
(**a**) MTS Criterion C; (**b**) Dantec devices used for the DIC process.

Three-point bending was applied as the main research method. Bending tests were held on the MTS Criterion C4 (MTS Systems GmbH, Berlin, Germany) shown in Figure 1. The research was performed in accordance with ASTM D790-10. The specimen was deformed until its outer surface cracked or until a maximum deformation of 5.0% was reached. The strain rate was 0.01 mm/min (according to procedure A from ASTM D790-10). The specimen was supported on two support points (placed on the longer side of the cross-section) and was loaded with a load lug positioned in the middle of the distance between the supports. Young’s modulus, conventional elastic limit (R_0.05_), and conventional yield point (R_0.2_) were calculated in accordance with ISO 178:2019. Furthermore, the digital-image-correlation (DIC) analysis was used to measure the distribution of deformation during the bending test. The measure was performed by means of Dantec Dynamics (Dantec, Ulm, Germany) devices and ISTRA 4D software shown in Figure 2.

Furthermore, in order to characterize the cracking propagation of the samples during the bending tests, fractographic research was performed. These observations were carried out using the KEYENCE VHX-7000 (Keyence, Osaka, Japan).

## 3. Results and Discussion

### 3.1. Flexural Properties—Bending Test

A series of tests were conducted using three different materials, including two composite materials. Samples of each material were tested in three series with different printing directions, as described in Section 2. Table 3 illustrates the results of the flexural strength σ_fM_ for the given samples, and the maximum value of deformation (due to the fact that all samples were cracked brittle, flexural strain at break ε_fB_ is equal to flexure strain at flexural strength ε_fM_).

**Figure 3:**
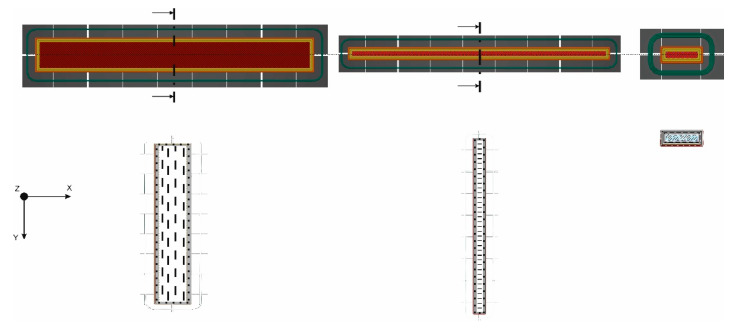
Orientation of fibers inside the tensile samples observed on the cross-section of samples processed according to different orientations, and schematic representation of the expected specimen microstructures [41].

The obtained results of the PA6 NB blend are typical for other, available polyamide-based materials acquired during the AM process [40]. The main role of the additions of PA-based blends dedicated for AM is to disturb the crystallization process, which, as a result, reduces the shrinkage stresses. The main principle of such a reduction is increasing the availability of PA molecular chains in a regular arrangement, especially during the cooling process [40]. In the case of the PA6 NB samples, the biggest plasticity of the material was registered, and the greatest strain during three-point bending was obtained for samples oriented along the Y direction (5.25%). The highest flexural strength was obtained for the PA6 CF15 material printed in the Y direction and its average value was 243.62 Mpa. Furthermore, the lowest value was calculated for the same material but produced in the Z direction, and it was 8.59 Mpa. PA6 CF15 was the most brittle material, and the lowest strain was obtained for samples produced in the Z direction (1.40%). Such dispersion of the obtained results in the case of the PA samples with carbon fibers is related to the AM orientation of samples which is additionally affected by the fiber distribution and void fraction. Such a phenomenon was analyzed by Badini et al. [41], where the authors revealed that fibers can be oriented differently inside the AM parts, mostly depending on the orientation in the substrate plated on the AM device. Such a phenomenon is shown in Figure 3.

The highest value of Young’s modulus was calculated for the PA6 CF15 material in the Y direction (11.65 Gpa), while the lowest value for PA6 GK10 was produced in the same Y direction (0.42 Gpa). The smallest obtained mechanical-properties values for the PA6 GK10 samples are a result of the nature of glass-microbeads’ addition. Their main role is to improve dielectric permittivity, reduce flammability, and increase thermal properties [42]. It is also worth noting that the worse mechanical properties caused by glass microbeads in the polyamide matrix are partially compensated by the enhanced shape and dimensional stability of AM parts [22,25]. Additionally, in the case of the usage of glass microbeads, Liang et al. [43] made studies on adding glass beads to the polymeric material, which led to an enhancement in Young’s modulus and impact strength when, at the same time, the yield strength was reduced as compared to the pure material. Such an enhancement in the case of the impact strength was attributed to the role of glass beads which block crazing at the interface that could not propagate further [25,43].

**Figure 4:**
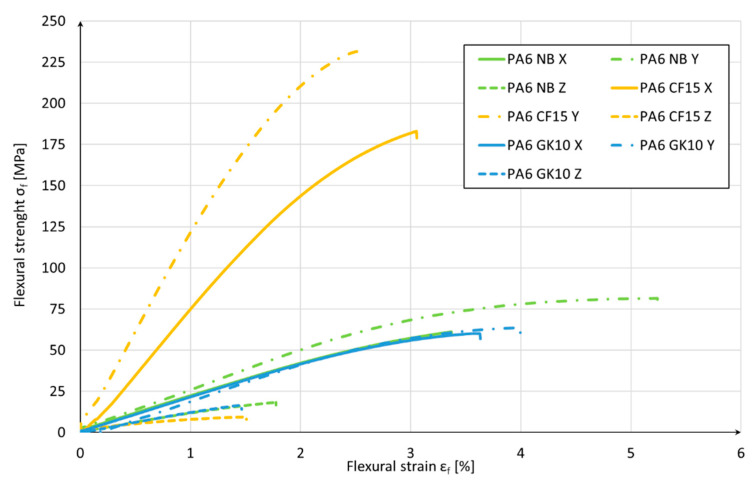
Curves of Flexural Stress (σ_fM_) versus Flexural Strain (ε_f_).

In Figure 4, a representative course of the bending test for the given materials and their orientation is shown.

Taking into account the test results from Table 3 and the diagram in Figure 4, comparing with the nonreinforced material (PA6 NB), it can be concluded that the use of carbon fibers positively affected the bending strength of the AM samples. At the same time, in the case of the reinforcement with the use of glass microbeads, the flexural strength significantly decreased. The main reason for the disproportion of mechanical properties in individual production directions is due to the fact that, in the case of the X and Y directions, the load was carried on by the material in the form of contour fibers and filling. Instead, in the case of samples produced in the Z direction, the load was carried on by the connection between the layers, and not by the material itself. These results show that the strength of the material is higher than that of the connection between the layers. 

### 3.2. Digital Image Correlation

**Figure 5:**
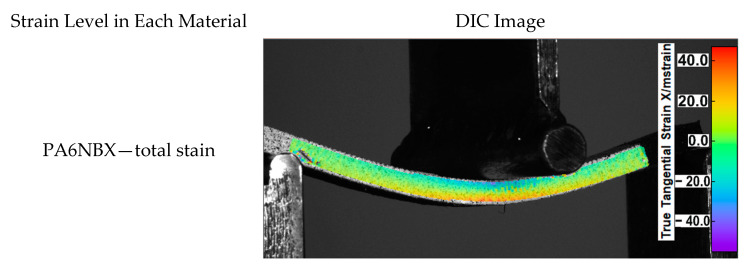

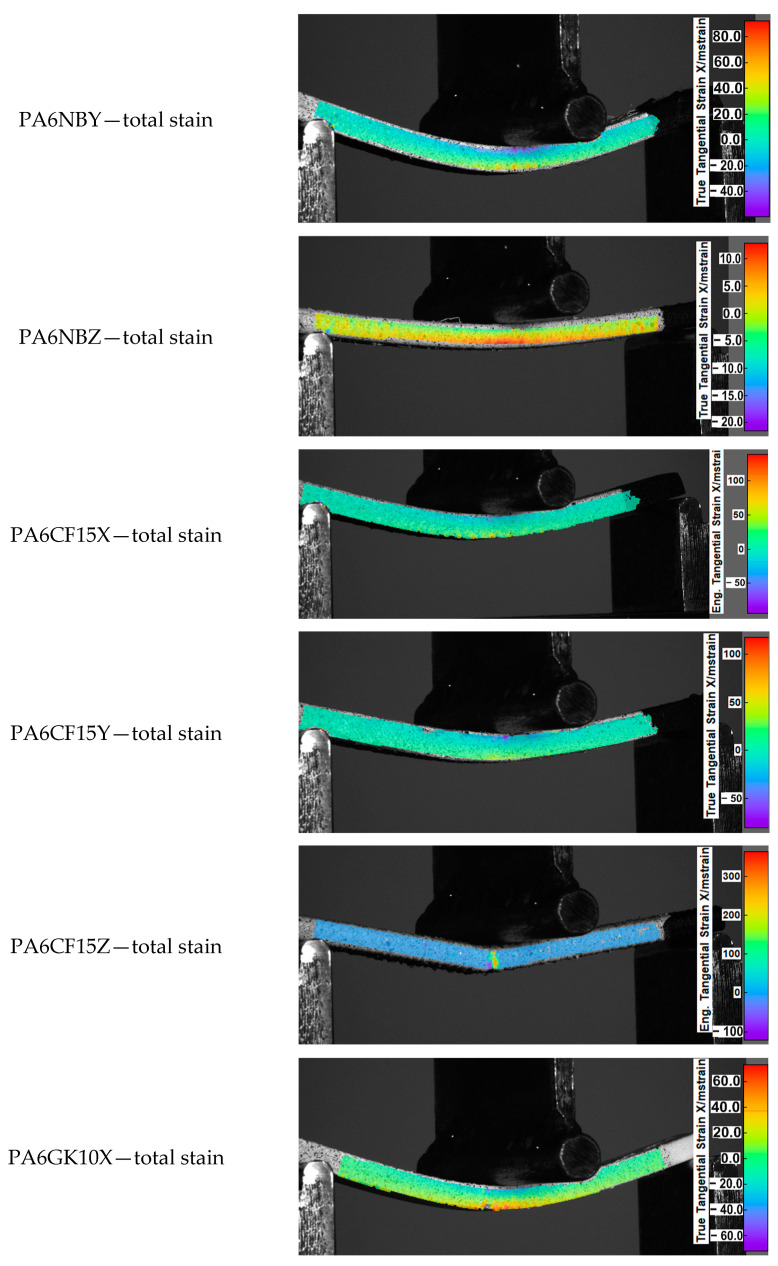

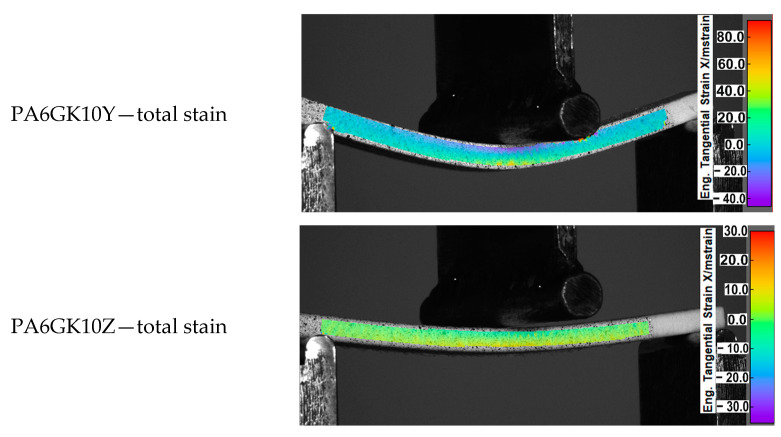
Strain levels registered via the DIC method.

During the bending tests, measurements were registered using the DIC method to show the distribution of displacements. Figure 5 shows the measurement results for a maximum strain for each material in different cases of build orientation.

The deformation distribution is uniform in each case in accordance with the theoretical assumptions. On the side of the load handle, deformations with negative values occur, so the material is compressed, while on the opposite side, it is stretched, which is typical in bending testing of conventionally made materials. The most significant differences in the deformation distribution between individual material types are visible for the Z direction. They are caused by loading the material parallel to the direction of joining individual layers and significant differences between the adhesive connection between the layers in each material. The PA6 NB and the PA GK10 materials did not break during the test. On the other hand, the sample made of PA6 CF15 material was fractured, which confirms the negative impact of the reinforcement on the strength of joints between the layers of the material. At the same time, in the case of the PA6 CF15 samples, the highest stiffness of the material was registered. Such a phenomenon could be observed when the fibers and the layer interfaces were parallel to the sample axis. Hence, two orientations of PA6 CF15 were found as the most resistant to bending (X and Y orientations). The principle of this issue is related to the positive influence of fibers on the improvement matrix characteristics (kind of polyamide matrix, matrix crystallinity, and porosity degree). For these reasons, the orientation of a carbon-fiber-reinforced part should be carefully considered when designing an AM process in order to assure proper fiber orientation to obtain the best possible mechanical properties in an exact application.

### 3.3. Fracture Analysis

**Figure 6:**
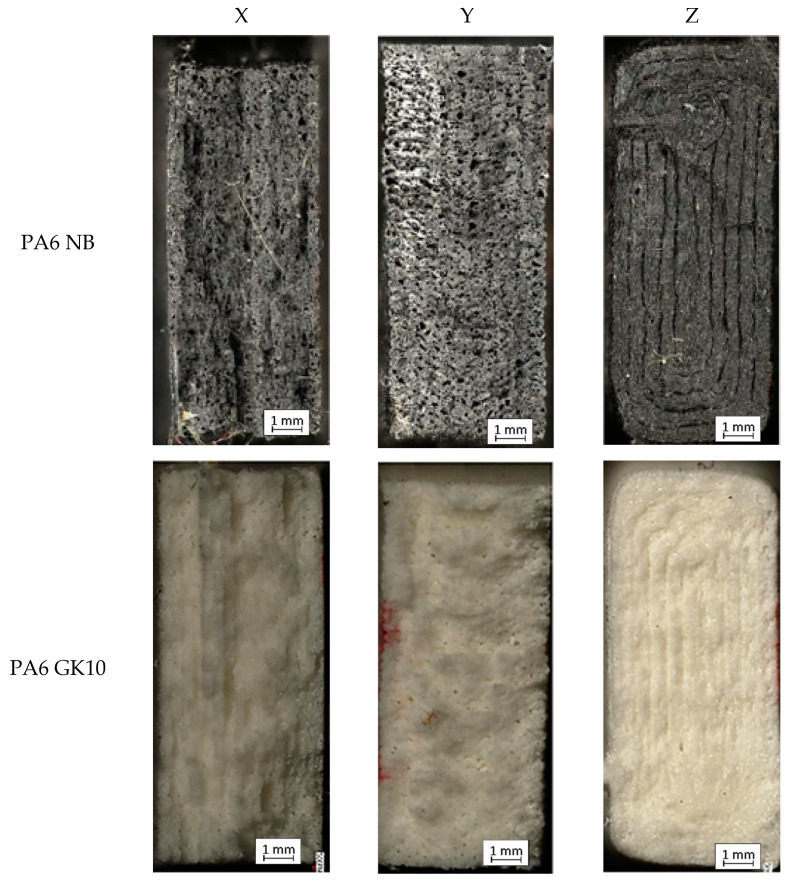

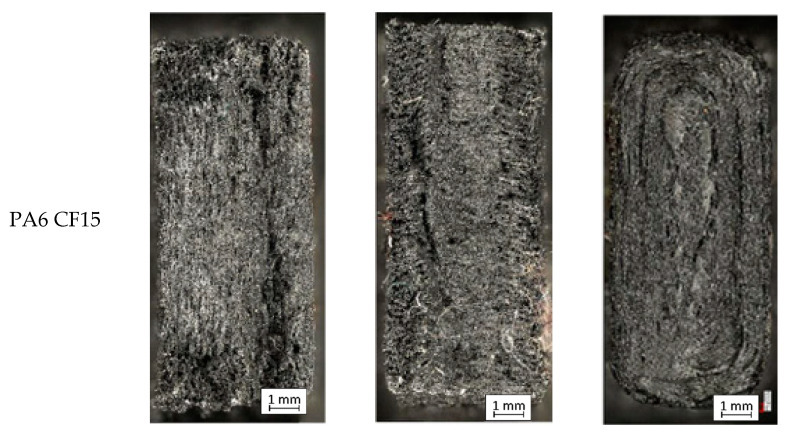
Fracture images of each sample.

After the bending tests, fractures of the samples were used for the microscopical examination to extend the research by fracture analysis. The result of a given process of each of the AM PA-based materials in three directions is depicted in Figure 6. In the case of pure PA6 NB samples manufactured along the X direction, a characteristic brittle-like fracture is observed (Figure 6, PA6 NB-X). This phenomenon was mostly caused by the visible voids in the material’s structure. In the case of the same material and samples oriented along the Y-axis, the fracture surface is quite similar; however, based on the bending-test results (Figure 5), the total flexural strength is visibly higher. Such behavior could be caused by a significant number of pores. During the material’s loading, the cracking mechanism went through the mentioned voids until there was total damage to the sample. In the case of the sample printed in the direction of the Z-axis, the sample was damaged at the point where the subsequent layers of the material were joined, transferring the lowest loads among all three printing directions.

When analyzing samples made of the PA6 GK10 material, similar breakthroughs are visible as in the case of the PA6 NB material samples. Samples made along the Y direction are characterized by a greater number of fracture planes, which confirms the highest bending strength observed during the bending tests. The breakthrough of the sample made along the Z direction looks very similar to the fracture of the corresponding PA6 NB material sample. The crack in the material went directly through the adhesion joint between the subsequent layers.

In the last material (PA6 CF15 composite), the fractures reveal the layered material structure and the brittle-like nature of the samples. The fractures of samples made along the X and Y axis look similar, but there are slight differences in the number of pores. Samples arranged along the Y-axis are characterized by a smaller number of pores, which translates into a greater percentage of solid material. Such a phenomenon results in greater material strength and changes the crack generation. Fractures of samples made along the X and Y-axis indicate structural heterogeneity connected with the local stress damming, which promoted a cracking initiation. It is possible to observe that cracking went through several layers, finally making an irregular, multiplanar crack. In the case of samples made along the Z-axis, the fracture has the same nature as two previous materials.

Furthermore, the arrangement of the layer interfaces (related to the orientation on the substrate plate) affects the mechanical behavior of AM parts during the fracture mechanism. That kind of phenomenon is related to the involvement of the debonding mechanism between the stacked layers [30]; it is also an answer for the worst mechanical properties of all samples produced along the Z-axis.

**Figure 7:**
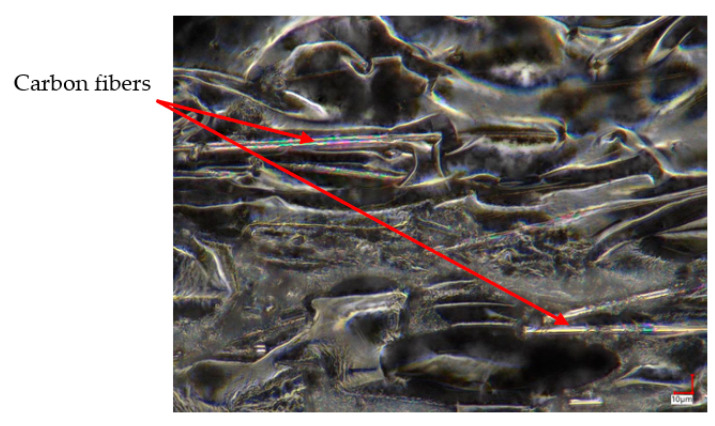
Microscope image of PA6 CF15X structure.

The positive results registered for two orientations (X and Y) are related to the decreased number of pores in the material volume. Such a phenomenon is illustrated in Figure 7, where all visible fibers are significantly surrounded by the base material.

The orientation of the carbon fibers is strictly related to the nozzle movement during each layer fabrication, as was already described and shown in Figure 4. As could be noticed, in the case of the PA6 CF15 samples’ fractures, such an orientation of fibers has a significant influence on void generation, which directly affects the mechanical properties. This is why the excellent properties of the PA6 CF15 samples can be explained by the dispersibility and the extrusion inducing a preferential orientation of carbon fiber in the PA matrix [30].

## 4. Conclusions

The conducted research results allowed us to determine the bending strength of polyamide-based materials, taking into account the influence of additives in the form of carbon fibers and glass microbeads compared to the pure, base material. The analysis of the research results enables the formulation of the following conclusions:

1. The PA6 CF15 material had the highest bending strength in the case of samples produced in the Y direction (243.62 MPa). However, carbon fibers negatively affected the overall ductility of the material.
2. The addition of glass microbeads reduced the bending strength in relation to the PA6 NB base material, regardless of the considered direction of sample manufacturing.
3. Measurements made by the DIC method revealed that the greatest differences in the deformation course took place in the case of the samples produced along the Z direction. They were especially visible in the case of the PA6 CF15 material, where the carbon fibers negatively affected the quality of the connection between successively deposited layers.
4. The addition of glass balls did not affect the course of material deformation.
5. Fracture analysis showed that the cracking course of the samples produced, irrespective of the direction, had a brittle-like character.
6. The samples produced along the Y-axis had a less complex fracture topography than the samples produced along the X-axis.
7. Regardless of the material type, in the orientation along the Z-axis, the cracks occurred at the joint between the successive layers.

In future research, the authors will try to reduce the presence of voids in the additively manufactured PA-based materials by means of heat treatment in vacuum conditions. A positive result of such postprocessing would be examined from the point of view of mechanical properties.

## Figures and Tables

**Table 1:** Properties of used materials [38].

Property	PA6 Neat Black	PA6 GK10	PA6 CF15
Density (g/cm^3^)	1.25	1.01	1.25
Tensile strength (MPa)	78	87	170
Extension at max. force (%)	4.4	2.7	2
Modulus of elasticity (GPa)	3.4	4.2	15

**Table 2:** Parameters of the FFF process suggested by the filament supplier.

Material	Filament Diameter (mm)	Nozzle Diameter (mm)	Table Temperature (°C)	Nozzle Temperature (°C)	Infill (%)	Number of Contours
PA6 NB	1.75	0.4	80	260	100	5
PA6GK10	1.75	0.4	80	260	100	5
PA6GK15	1.75	0.4	80	260	100	5

**Table 3:** Registered bending properties of all tested samples.

Material		Average of Flexural Strength σ_fM_ (MPa)	Standard Deviation (Mpa)	Average of Flexural Strain ε_fB_ (%)	Standard Deviation (%)	Young’s Modulus E (Gpa)	Standard Deviation (Mpa)
PA6 NB	X	66.75	4.94	4.87	1.32	2.97	0.20
	Y	81.39	1.64	5.25	0.01	1.96	0.45
	Z	23.54	0.70	1.73	0.09	1.11	0.06
PA6 CF15	X	184.18	3.44	3.26	0.18	6.12	0.22
	Y	243.62	10.85	2.53	0.16	11.65	1.41
	Z	8.59	3.65	1.40	0.30	0.92	0.26
PA6 GK10	X	60.10	1.29	3.87	0.21	2.75	0.44
	Y	64.05	0.55	3.95	0.11	0.42	0.34
	Z	16.76	0.44	1.56	0.14	1.09	0.14

## Data Availability

Not applicable.
